# Optimizing drug combination and mechanism analysis based on risk pathway crosstalk in pan cancer

**DOI:** 10.1038/s41597-024-02915-y

**Published:** 2024-01-16

**Authors:** Congxue Hu, Wanqi Mi, Feng Li, Lun Zhu, Qi Ou, Maohao Li, Tengyue Li, Yuheng Ma, Yunpeng Zhang, Yingqi Xu

**Affiliations:** 1 College of Bioinformatics Science and Technology, Harbin Medical University, Harbin, 150081 China; 2 Department of Pharmacy, Inner Mongolia Medical University, Jinshan Development Zone, Hohhot, 010100 China

**Keywords:** Computational biology and bioinformatics, Cancer

## Abstract

Combination therapy can greatly improve the efficacy of cancer treatment, so identifying the most effective drug combination and interaction can accelerate the development of combination therapy. Here we developed a computational network biological approach to identify the effective drug which inhibition risk pathway crosstalk of cancer, and then filtrated and optimized the drug combination for cancer treatment. We integrated high-throughput data concerning pan-cancer and drugs to construct miRNA-mediated crosstalk networks among cancer pathways and further construct networks for therapeutic drug. Screening by drug combination method, we obtained 687 optimized drug combinations of 83 first-line anticancer drugs in pan-cancer. Next, we analyzed drug combination mechanism, and confirmed that the targets of cancer-specific crosstalk network in drug combination were closely related to cancer prognosis by survival analysis. Finally, we save all the results to a webpage for query (http://bio-bigdata.hrbmu.edu.cn/oDrugCP/). In conclusion, our study provided an effective method for screening precise drug combinations for various cancer treatments, which may have important scientific significance and clinical application value for tumor treatment.

## Introduction

Despite significant advancements made in the treatment of cancer during the past several decades, it remains one of the leading causes of death worldwide killing approximately 9.9 million people annually. The occurrence and development of cancer is the result of the interaction of multiple factors, multiple molecules (such as genes, transcription factors, proteins, microRNAs(miRNAs)), and complex regulatory networks between molecules. So, the crosstalk between pathways in cancer, as well as the function and interaction of biological pathways mediated by regulatory molecules such as miRNA through target genes, make it impossible to achieve the ideal effect of single drug in cancer treatment, and it is easy to develop drug resistance and tumor recurrence^1^. At present, chemotherapy is an indispensable means of cancer treatment, but the effect of chemotherapy is not ideal. In addition to drug resistance, there are also problems such as narrow treatment window and serious adverse reactions^2^. In consequence, exploring the interaction mechanism of miRNA-mediated pathways in malignant tumors and developing and designing algorithms based on this interaction mechanism to optimize effective drug combination have important practical guiding significance for cancer treatment.

In recent years, an increasing number of studies have developed bioinformatics algorithms based on computational theoretical models to predict effective drug combinations. These methods mainly focus on the interaction between chemical structures of drugs^3^, the protein interaction between drug target genes^4^, or the pathway of two drugs to identify effective drug combinations^5^. These bioinformatics-based computational methods have helped researchers successfully identify a variety of effective drug combinations. With the deepening of research, researchers have gradually realized that the occurrence and development of tumors are regulated by multiple factors and layers. More and more studies have shown that drugs not only produce therapeutic effects by acting on genes, but also can affect miRNAs to inhibit tumors^6,7^.

**Fig. 1:**
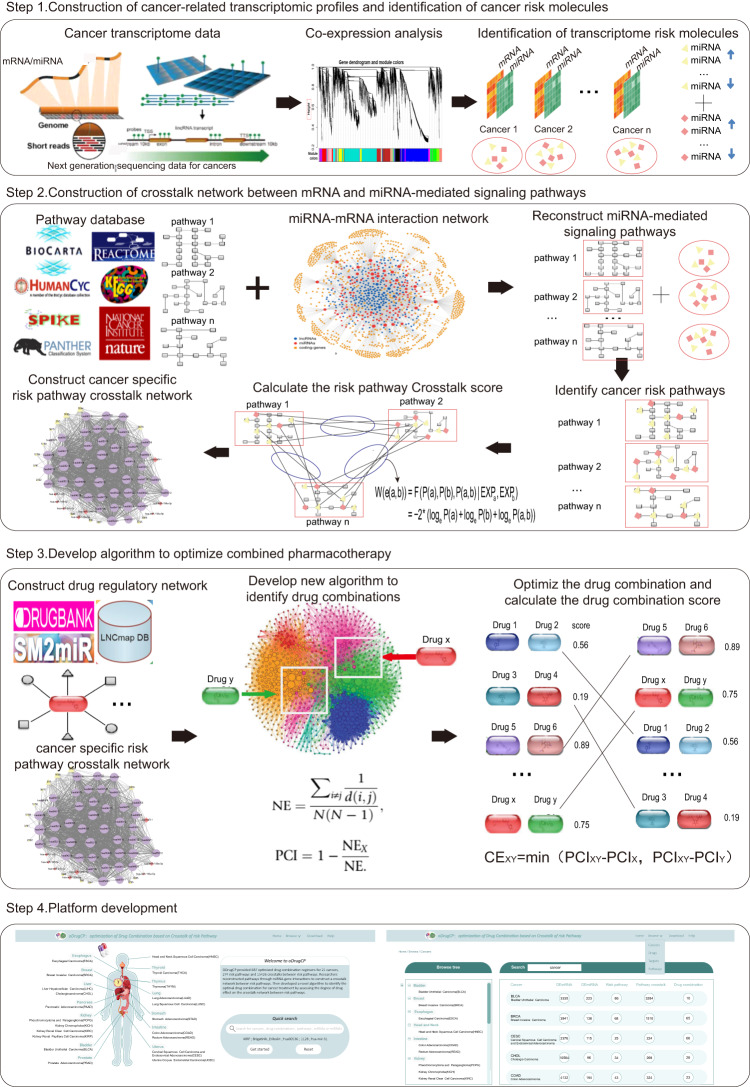
Workflow of this research.

In this paper, we aim to regulate the pathway crosstalk in cancer through combination anti-cancer drugs to maximize the therapeutic effect and prolong the survival of patients. Inspired by the challenge, we constructed crosstalk between risk pathways by identifying critical mRNAs and miRNAs -mediated risk pathways in cancer. Furthermore, we developed new calculation methods to identify the most effective drug combinations through specific crosstalk between the risk pathways that act on mRNAs and miRNA co-regulated. Finally, we developed a query and analysis platform for the combination of anticancer drugs which provides effective and accurate treatment strategies for cancer (Fig. 1).

## Results

### Risk mRNA and miRNA landscape in pan-cancer

**Fig. 2:**
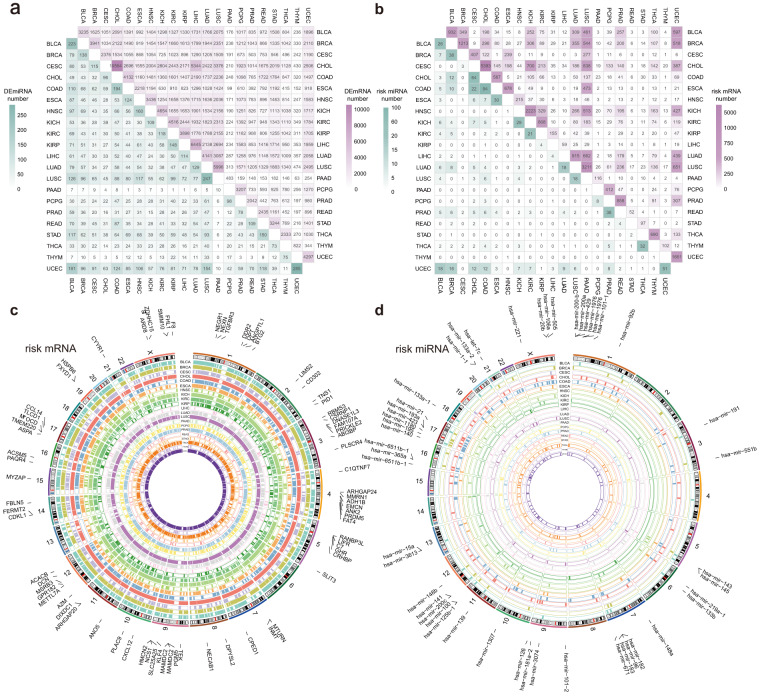
Risk mRNAs and miRNAs in 21 cancers. (**a**) The number of differential mRNA (purple, upper right) and differential miRNA (green, left lower) in each cancer and the number of intersections between them. (**b**) The number of risk mRNA (purple, upper right) and risk miRNA (green, left lower) in each cancer and the number of intersections between them. (**b**) Position of 21 cancers’ risk mRNA on chromosome. Each circle represents the risk mRNA for one cancer. (**d**) Position of 21 cancers’ risk miRNA on chromosome. Each circle represents the risk miRNA for one cancer.

In order to accurately grasp the pathogenesis of cancer and find the risk factors of different cancers, we first performed differential analysis on cancer samples and normal samples by unpaired Student’s t-test and fold change method. In total, we obtained 16197 differential mRNAs and 552 differential miRNAs in 21 cancers (Fig. 2a). However, by comparison, we found that there are a large number of identical differential mRNAs and miRNAs among different cancers, indicating that the differential factors are universal and cannot effectively distinguish the signatures of different cancers (Fig. 2a, Supplementary Table 1).

Therefore, to further identify the key risk factors for different cancers, co-expression analysis of mRNA and miRNA was used to identify the sets with highly synergistic changes in expression, and risk mRNAs and miRNAs were screened according to the degree of correlation between the sets and phenotypes (cancer and normal). A total of 11343 risk mRNAs and 278 risk miRNAs were obtained (Fig. 2b, Supplementary Table 2). We found that the proportion of cancers sharing risk factors was much lower than the proportion sharing differential factors (Fig. 2b). For example, there were 3235 differentially expressed mRNAs in BLCA and 2376 in CESC, with 1051 intersections between them, but there were 932 risk mRNAs in BLCA and 407 in CESC, and only 2 intersections. Furthermore, we obtained the chromosomal positions of risk mRNA and risk miRNA, and found that in some cancers, risk mRNA and risk miRNA tended to appear on the same chromosomal position, which may suggest that these cancers have similar risk characteristics (Fig. 2c-d). Thus, risk mRNAs and miRNAs could be included in subsequent analyses as signatures of each cancer.

### Identifying risk pathways in pan-cancer

**Fig. 3:**
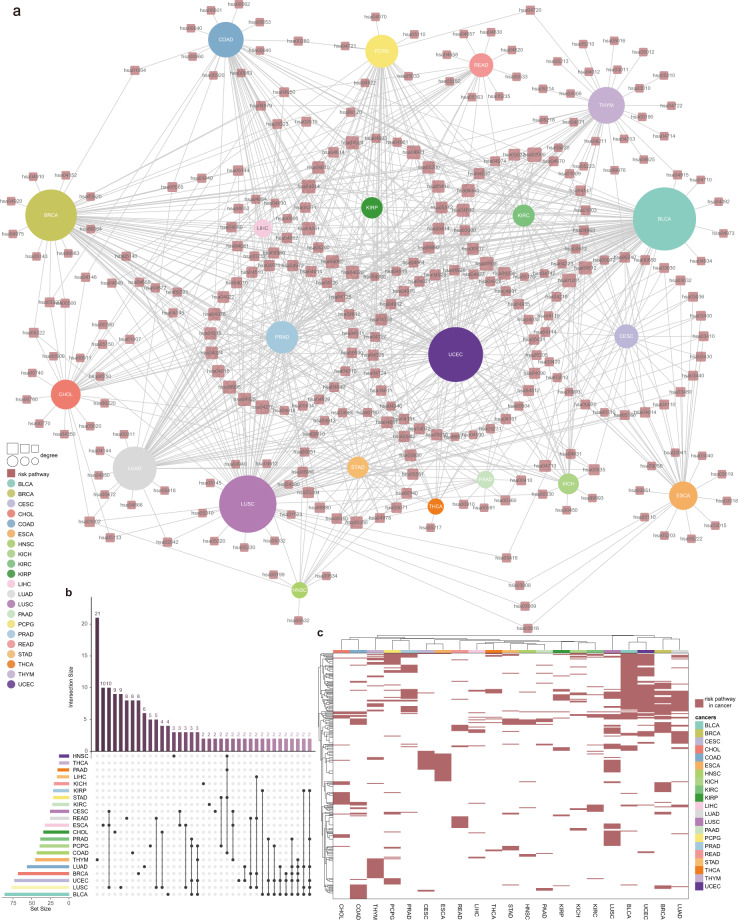
Risk pathways of 21 cancers. (**a**) Risk pathway network in pan-cancer. (**b**) The bar plot on the left represents the number of risk pathways in each cancer. The dot plot on the lower right represents multiple sets of risk pathways. A single dot indicates that the risk pathways in the set are only present in one cancer, and a line between two or more dots indicates that the risk pathways in multiple cancers overlap. Only sets with the number of paths greater than 2 are shown here. The bar chart at the top right shows the number of pathways in the sets. (**c**) Risk pathway heatmap in pan-cancer. Hierarchical clustering was performed on the rows (risk pathways) and columns (cancer) of the heatmap.

Occurrence of cancers are affected by multiple factors and pathways. In order to reflect the molecular mechanism of cancer veritably and accurately, we used the methods that we developed previously to reconstruct all biological pathways among KEGG(https://www.genome.jp/kegg/). miRNAs were added into the signaling pathway by the targeting relationship between miRNA and target mRNA, which constitutes a more abundant signaling pathway. Based on hypergeometric enrichment analysis of cancer risk mRNAs and risk miRNAs, and with significant P < 0.05 as the threshold, we identified risk pathways of each cancer. Totally, there were 274 cancer risk pathways in 21 cancer types (Fig. 3, Supplementary Table 3). Next, we constructed a regulatory network of cancer risk pathways, and the results showed that only one third of risk pathways were cancer-specific risk pathways, and most were shared by multiple cancers (Fig. 3a-b). These cancer-specific risk pathways may be responsible for the different clinical outcomes and medication differences in pan-cancer. Among them, THYM had the greatest number of cancer-specific risk pathways (Fig. 3a-b). Thymoma often has specific manifestations and is associated with a variety of paraneoplastic syndromes. Some studies have also found that patients with thymoma have an increased risk of second primary tumors^8^. Furthermore, LUSC, ESCA and CESC have the same 10 risk pathways, which may be related to the phenomenon that most of these three cancers are present as squamous cell carcinoma (Fig. 3b). In addition, it was also found that risk pathways such as transporters^9^, glycosaminoglycan binding proteins^10^, protein kinases^11^, vascular smooth muscle contraction^12^, cell adhesion molecules (CAMs)^13^, gastric acid secretion^14^ in several cancers have been confirmed to be closely related to the occurrence and development of cancer(Fig. 3a). In particular, BLCA, BRCA, LUAD, LUSC and UCEC share similar risk pathway patterns, suggesting that these cancers may share similar drug treatment modalities (Fig. 3c).

### Constructing risk pathway crosstalk network in pan-cancer

**Fig. 4:**
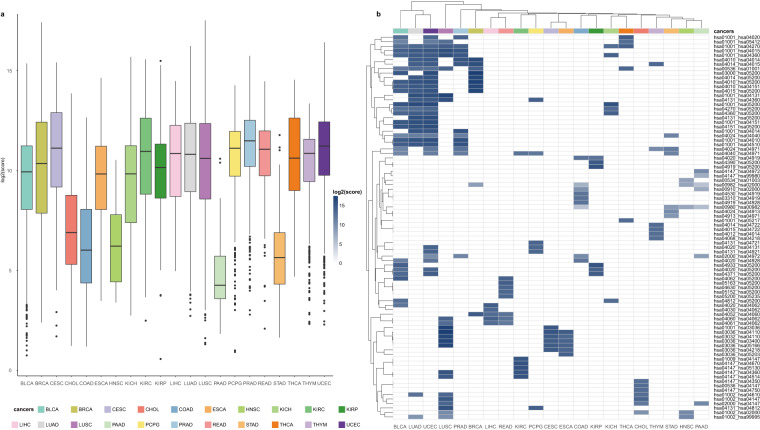
Pathway crosstalk between 21 cancers’ risk pathways. (**a**) Boxplot of log2 pathway crosstalk score for 21 cancers. (**b**) Heatmap of log2 score in top 5 pathway crosstalk for 21 cancers.

**Fig. 5:**
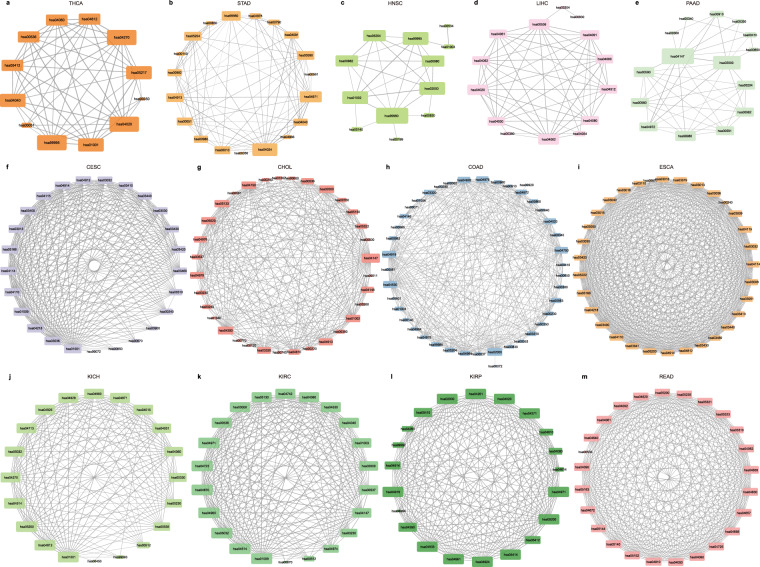
Pathway crosstalk networks in 13 cancers.

During the occurrence and development of cancer, there are certain crosstalk between different biological pathways. Therefore, exploring the crosstalk between cancer-related biological pathways is an important link in the analysis of cancer. Here, we further investigated pan-cancer from the functional level to dissect the mechanism of cancer occurrence and development. We constructed and quantified the crosstalk of cancer risk pathways by calculating the correlation values of mRNAs and miRNAs between risk pathways and the degree of difference between cancer samples and normal samples. Crosstalk between risk pathways were constructed for 21 cancer types respectively (Fig. 4a, Supplementary Table 4). Among them, five cancers had low pathway crosstalk scores, and the remaining cancers had high crosstalk activity between pathways, indicating the complexity and heterogeneity of cancer molecular mechanisms. Furthermore, protein kinases and exosomes appear in the crosstalk of multiple cancers (Fig. 4b, Fig. 5). Recent studies indicate that the complex crosstalk between the phosphatidylinositol-3-kinase-protein kinaseB-mammalian target of rapamycin pathway and multiple interacting cell signaling cascades can further promote prostate cancer progression^15^. Meanwhile, the exosomes derived from chronic myeloid leukemia cells contained a cytokine, TGFβ1, which binds to the TGFβ1 receptor on the leukemia cells, thereby promoting tumor growth through activation of extracellular signal-regulated kinase, protein kinase, and anti-apoptotic pathways in the producer cells^16^. These results suggest that these cancer-specific risk pathway crosstalk networks may be one of the molecular mechanisms leading to different clinical outcomes in various cancers, which will help us to understand the similarities and differences between cancers and point out new ways to optimize cancer treatment.

### Optimizing drug combination for cancer treatment

**Fig. 6:**
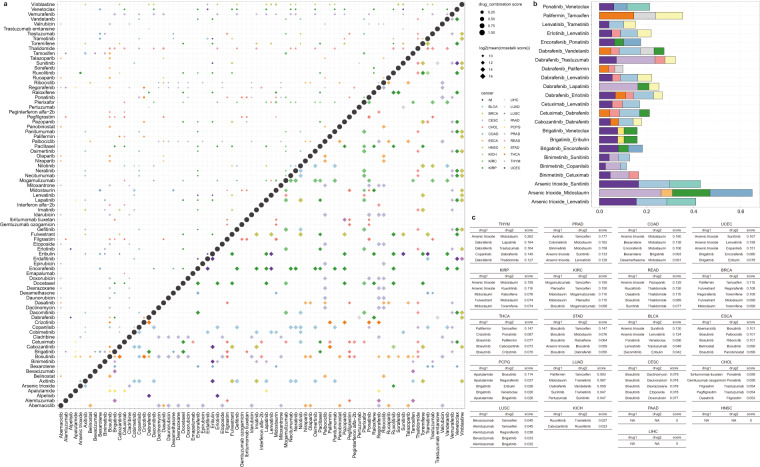
Optimal combination plan for 21 cancers’ treatment drugs. (**a**) Drug combinations that appear in only one cancer. The size of the dot represents the drug combination score. The size of the diamond represents the log2 of mean all crosstalk pathways involved in the drug combination. The color represents cancer. (**b**) Optimal drug combination plans present in more than three cancers. The X-axis is the drug combination score, and the Y-axis is the drug combination plan. The color of the bar represents cancer. (**c**) Top 5 drug combinations in pan-cancer.

First, target information of 191 anticancer drugs in the NCI database was obtained from DrugBank(https://go.drugbank.com/) and SM2miR database (http://www.jianglab.cn/SM2miR/). We then filtered candidate therapeutic drugs for each cancer by evaluating their effect on the risk pathway crosstalk. Finally, in total 83 candidate drugs were obtained for 21 cancers. Next, we combined the 83 drugs in pairs, calculated the crosstalk score after removing the combined drug target, quantified the extent to which each combination disrupted the crosstalk between cancer risk pathways, and then compared the damage values obtained from these combination drugs with the sum of the damage values of the two drugs in the combination. We selected a combination with a combination destruction score greater than the sum of the two drug destruction scores. Ultimately, 687 combination regimens of 18 cancers were available, of which 400 were cancer-specific (Fig. 6a, Supplementary Table 5), 104 were shared between two cancers, and 23 could be used to treat three or more cancers (Fig. 6b). More importantly, the combination of drugs can be ranked according to the score in each cancer to select the optimal combination of drugs for cancer treatment (Fig. 6c).

In our drugs’ optimization results, Vinblastine was found to be available in combination with other drugs to specifically treat BRCA, while Vemurafenib was only effective in THYM (Fig. 6A. top left). Research had shown that the combined treatment of BRCA cells with actinomycin-D or vinblastine and brefeldin A or golgicide A, two disrupting agents of the ARF1 function, resulting in decreased cell proliferation and cell migration, as well as in increased apoptosis^17^. However, there was no reference to the role of vemurafenib in the treatment of THYM. Only studies verified that the addition of vemurafenib to cetuximab and irinotecan can improve progression-free survival and treatment response of colorectal cancer patient^18^. Meanwhile, vemurafenib monotherapy was effective for treating patients with BrAFV600-mutated NSCLC^19^. These studies showed that vemurafenib could provide a therapeutic possibility for THYM. Moreover, the crosstalks of risk pathways involved in all optimized drug combinations were very strong (Fig. 6a lower right).

We also found that the combination of drugs, including binimetinib and dabrafenib, with other drugs can highly disrupt inter-pathway crosstalk in multiple cancers (Fig. 6b). Vemurafenib-cobimetanib, dabrafenib -trametinib, and encorafenib -binimetinib were the FDA-approved combinations for the treatment of BRAFV600E melanomas^20^. And combination of cisplatin, pemetrexed and binimetinib presented no unexpected toxicity, may be used to treat NSCLC^21^. Dabrafenib-trametinib was the first regimen demonstrated to have robust clinical activity in BRAF V600E-mutated anaplastic thyroid cancer and was well tolerated^22^. These results indicated that some drug combinations had commonalities and pleiotropy in cancer treatment.

**Fig. 7:**
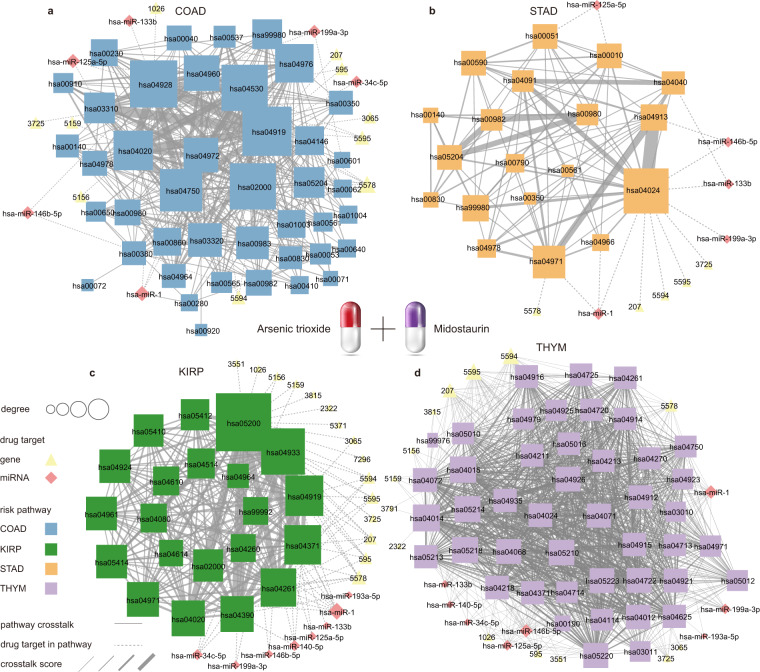
Effects of asides trioxide and midostaurin on the crosstalk network of specific risk pathways for (**a**) COAD; (**b**) STAD; (**c**) KIRP; (**d**) THYM.

In addition, the drug combination of asides trioxide and midostaurin had high crosstalk scores in the four cancers (Fig. 6b). We found that this drug combination can be achieved by targeting the same targets JUN, PRKCA, MAPK1, MAPK3, hsa-mir-1, hsa-mir-12in addition5a, hsa-mir-133b, hsa-mir-146b, hsa-mir-199a and other specific targets, respectively, act on the COAD, KIRP, STAD, THYM - specific crosstalk network, and then obtain very high scores (Fig. 7). Although this drug combination had the same target in all four cancers, the pathways it perturbed in different cancers were inconsistent. This illustrated the cancer specificity of drug combination regulatory mechanism.

**Fig. 8:**
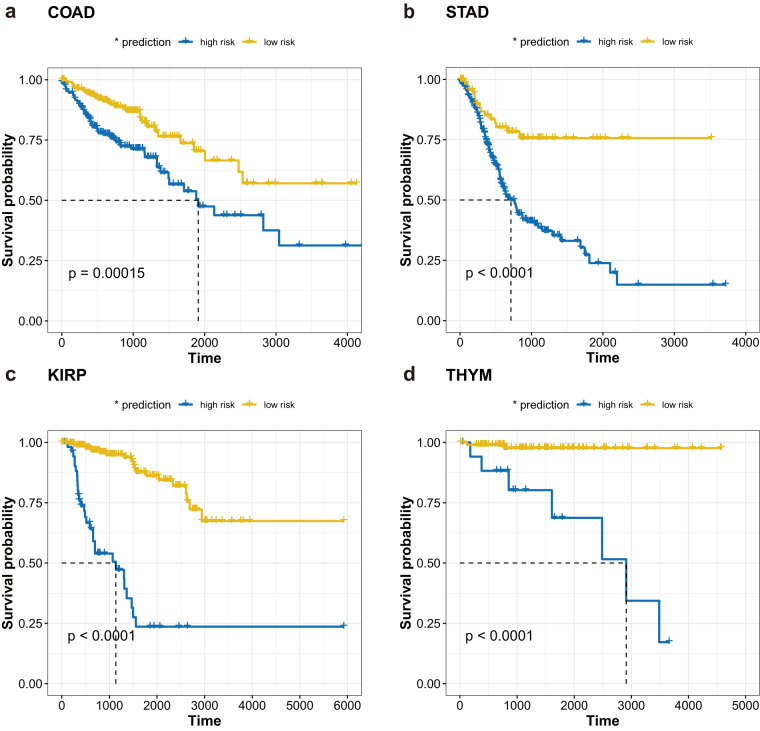
Survival curves of drug combination targets for four cancers. (**a**) COAD; (**b**) STAD; (**c**) KIRP; (**d**) THYM.

To further analyze the clinical value of this drug combination, we obtained clinical survival information from patients in four cancers and evaluated the impact of drug target signaling on patient survival. According to the expression of drug target signatures, patients were divided into shorter survival group and longer survival group. There was a significant difference in survival time between these two groups. The results confirmed that, the targets in the four cancer-specific crosstalk networks were closely related to the prognosis of the cancer (Fig. 8).

In order to promote the research of drug combination therapy for cancer, we put all the research results on the web page(http://bio-bigdata.hrbmu.edu.cn/oDrugCP/). The Web page provides a user-friendly interface mainly consisting of three modules: Search, Browse and Download. The quick search enables users to filter entries with one keyword of interest, such as a cancer, or drug name. Also, an advanced search is provided in ‘Search’ page for more specific requirements. The users can input interested cancer and drug combination at the same time to obtain involved drug target and pathway. In ‘Browse’ page, all cancers and drug combinations in our research are listed, respectively, for users to query. The search and browse results can be freely downloaded. Furthermore, ‘Help’ page contains detailed guidance for users.

## Discussion

In recent decades, with the deepening understanding of the complexity of malignant tumors, targeted drugs have gradually become the main means of treating cancer because of their high specificity and remarkable curative effect on various malignant tumors. However, drug resistance has become a major challenge for anticancer therapy. Tumors often develop resistance to monotherapy due to the heterogeneity of tumor responses to drugs. To overcome the limitations of single drug therapy, combination therapy has been proposed as a promising therapeutic strategy. In combination therapy, multiple drugs can act on multiple targets and pathways, which greatly improves the therapeutic effect, slows the drug resistance of tumors, and reduces the toxicity of drugs. However, the current application of drugs for combination therapy is very limited and only a part of the huge treatment space. In this study, we identified potential anti-cancer synergistic drug combinations in 21 cancers based on the effects of risk mRNAs and risk miRNAs on dysfunctional pathways and the crosstalk system between the pathway networks. It provides some new options for drug selection in pan-cancer patients.

In this study, we integrated human cancer transcriptome data, pathway data, and drug target information to develop a method for predicting synergistic anti-cancer drug combinations. First, we identified cancer-associated risk mRNAs and risk miRNAs using differential analysis and co-expression analysis. Next, we added miRNAs to reconstruct all biological pathways to identify risk pathways for each cancer by hypergeometric analysis of risk factors. Finally, we constructed and quantified the crosstalk of cancer risk pathways and then identified and optimized cancer drug treatment by evaluating the disruption of pathway crosstalk by all marketed anticancer drugs. Analysis of the overall properties of risk genes revealed that they broadly affect a variety of biological functions. In addition, risk pathway crosstalk suggests differential regulatory mechanisms in different cancers. More importantly, based on our approach, not only drug combinations that are widely used in a variety of cancers, but also some cancer-specific drug combinations were screened. The survival analysis of drug combination targets highlights its potential for clinical application as a prognostic biomarker and suggests its role in cancer therapy.

Currently, several methods have been proposed to optimize drug combination for human cancers. For example, Peiran *et al*. proposed a Graph Convolutional Network (GCN) model to predict synergistic drug combinations in particular cancer cell lines^23^. Furthermore, Malas *et al*. prioritized drugs using the semantic information between drug and disease concepts^24^. Comparing with these methods, our study has some unique features. First, we considered the role of miRNAs in our approach. Second, our study optimized anticancer drugs by measuring their effects for mediating the crosstalk between risk pathways, which was an important molecular mechanism in the initiation and progression of human cancers. Tertiary, we optimized candidate drugs for 21 cancers, which may further promote the precise use of drugs for human cancer. Last but not least, We optimized the combination of 191 clinical first-line anticancer drugs, and the obtained results can be applied clinically, rather than a vague and meaningless study.

Our research has some unique aspects. First, we reconstructed biological pathways and added miRNAs to the pathways. Studies have shown that miRNAs are closely related to the occurrence and development of cancer and drug sensitivity. The reconstructed pathway takes into account the regulation of upstream and downstream genes, and can provide a broader understanding of the mechanism of action of the response pathway, which provides a good foundation for the subsequent analysis of the mechanism of cancer. Second, we focus on the crosstalk between pathways as an important factor affecting cancer treatment, and our method identifies drug combinations that have a great impact on the inhibition of crosstalk between pathways, providing new insights into the precise treatment of cancer. In conclusion, our study offers an effective way to screen precision drug combination for various cancers’ treatment. We also dissected the mechanism of optimal therapeutic drugs, which may provide novel insight into the precise treatment of cancer and promote researches on the mechanisms of action of drugs.

In order to comprehensively evaluate the effectiveness of this method in predicting drug combination, it was compared with the existing advanced methods. Patricia Jaaks *et al*. evaluated the synergy of drugs based on potency (ΔIC50) and efficacy(ΔEmax) to identify synergistic drug combinations^25^. The results showed that 12 of our predicted drug combinations appeared in the drug combinations identified by Patricia Jaaks *et al*. (Supplementary Figure 1), indicating the effectiveness of our method in predicting drug combinations. And our approach takes into account not only RNA, but also microRNA. Currently, there are drugs targeting microRNA on the market, which also suggests that it is necessary to use microRNA as a factor in drug screening. Our method comprehensively considers RNA, microRNA and pathway, and has high reliability. In addition, we validated our results using drug combination data stored in three databases, including DrugComb(https://drugcomb.fimm.fi/)^26^, DrugCombDB(http://drugcombdb.denglab.org/main)^27^ and SYNERGxDB(https://www.synergxdb.ca/)^28^. The results showed a high degree of coincidence between our predicted outcomes and the drug combinations stored in the database. Of the 687 drug combinations, 223 were confirmed in these databases, which stored experimentally proven, documented, and external databases, with 33 in DrugComb and 207 in DrugCombDB (Supplementary Figure 1). 68 drug combinations are included in SYNERGxDB.

In addition to miRNAs, lncRNAs and ceRNAs can also mediate pathways by co-regulating with mRNAs, however, drugs targeting microRNA have been approved and certified by the FDA, we only investigated miRNAs in this study.

## Methods

### miRNA and mRNA expression profiles from pan-cancer samples

We obtained mRNA expression data and matched miRNA expression data for tumor individuals from the TCGA (The Cancer Genome Atlas) database (http://tcga-data.nci.nih.gov/)^29^. In this study, we selected tumor types containing normal samples for further analysis. Finally, mRNA expression datasets and matched miRNA expression datasets of 7660 tumor individuals and 620 normal samples from 21 cancer types (BLCA(Bladder Urothelial Carcinoma), BRCA(breast cancer), CESC(Cervical Squamous Cell Carcinoma and Endocervical Adenocarcinoma), CHOL(Cholangiocarcinoma), COAD(Colon Adenocarcinoma), ESCA(Esophageal Carcinoma), HNSC(Head and Neck Squamous Cell Carcinoma), KICH(Kidney Chromophobe), KIRC(Kidney Renal Clear Cell Carcinoma), KIRP(Kidney Renal Papillary Cell Carcinoma), LIHC(Liver Hepatocellular Carcinoma), LUAD(Lung Adenocarcinoma), LUSC(Lung Squamous Cell Carcinoma), PAAD(Pancreatic Adenocarcinoma), PCPG(Pheochromocytoma and Paraganglioma), PRAD(Prostate Adenocarcinoma), READ(Rectum Adenocarcinoma), STAD(Stomach Adenocarcinoma), THCA(Thyroid Carcinoma), THYM(Thymoma), UCEC(Uterine Corpus Endometrial Carcinoma)) were obtained.

### miRNA-Target mRNAs relationship Data

In this study, we collected experimentally verified miRNA-target interactions from four known public databases: miRTarBase(https://mirtarbase.cuhk.edu.cn/~miRTarBase/miRTarBase_2022/php/index.php)^30^, mir2Disease(http://www.mir2disease.org/)^31^, miRecords^32^ (V4.0)(http://miRecords.umn.edu/miRecords), and TarBase^33^ (V6.0)(http://www.diana.pcbi.upenn.edu/tarbase), extracted and integrated human-related miRNA-target mRNA relationships to obtain a more comprehensive dataset. Finally, a total of 57,863 miRNA-target relationships were collected, including 579 miRNAs and 14,652 target mRNAs, for further analysis.

### PPI Network and Pathway Data

The protein-protein Interaction (PPI) network data used in this study came from HPRD (Human Protein Reference Database) (http://www.hprd.org/)^34^. Interactions stored in HPRD were mainly from experimental validation and text mining, and a total of 34165 protein-protein interactions were obtained in this study. The pathway data used for functional analysis in this study were obtained from the Kyoto Encyclopedia of mRNAs and Genomes (KEGG) database^35^.

### Drug and Drug Target Data

By using DrugBank(https://go.drugbank.com/)^36^ and SM2miR(http://www.jianglab.cn/SM2miR/)^37^ databases, we obtained drugs and their corresponding target mRNAs and target miRNA respectively. Meanwhile, we only selected anti-cancer drugs in Cancer Drugs of NCI (NATIONAL CANCER INSTITUTE) (https://www.cancer.gov/about-cancer/treatment/drugs). A total of 191 drugs. DrugBank database integrates chemical and biological information resources, providing detailed drug data and drug target data. The SM2miR database, which records the interactions between drugs and miRNA in multiple species, provides a comprehensive overview of the effects of small molecules on miRNA expression.

### Reconstructed KEGG Pathway Graphs

The reconstructed KEGG (Kyoto Encyclopedia of mRNAs and Genomes)(https://www.genome.jp/kegg/) pathway igraph contains mRNAs, miRNAs and real biological pathways. We first collected 406 KEGG pathway data and converted them into an undirected graph with mRNAs as nodes and interactions between mRNAs as edges using our previously developed R package iSubpathway Miner^38^. We then reconstructed these pathways by linking miRNAs to these pathways by integrating miRNA-target relationship and pathway data. In other words, if the target mRNAs of a particular miRNA were significantly enriched in a pathway, the miRNA was linked to that pathway by linking to the target mRNA in that pathway. The significance of enrichment was assessed using the hypergeometric test. The formula is as follows:

$${\rm{P}}=1-\mathop{\sum }\limits_{{\rm{k}}=0}^{{\rm{m}}}\frac{(\begin{array}{c}{\rm{n}}\\ {\rm{k}}\end{array})(\begin{array}{c}{\rm{N}}-{\rm{n}}\\ {\rm{M}}-{\rm{k}}\end{array})}{(\begin{array}{c}{\rm{N}}\\ {\rm{M}}\end{array})}$$

where N represents the number of background mRNAs (all genome-wide mRNAs), M is the number of mRNAs involved in a given pathway, n is the number of target mRNAs for a specific miRNA, and m is the number of miRNA target mRNAs annotated in the given pathway.We chose miRNAs with P < 0.05 in the enrichment analysis as pathway miRNAs that could be added to the pathway, and eventually reconstructed the pathway.

### Identification of Risk mRNAs and miRNAs Related to the 21 Cancer types

For each cancer type, we identified significantly differentially expressed mRNA and miRNA based on expression data from cancer and normal samples. We evaluated differentially expressed mRNAs/miRNAs using both an unpaired Student’s t-test and fold change method. Then, the significance p-value of the t-test was corrected by the Benjamini-Hochberg multiple test^39^ to obtain the false discovery rate (FDR) value. Finally, we choose $\mathrm{FDR} < 0.01,{\log }_{2}| \mathrm{FC}|  > 2$ and P < 0.05 as thresholds to identify differentially expressed mRNAs/miRNAs, these differentially expressed mRNAs/miRNAs are cancer-related mRNAs/miRNAs, which we define as cancer risk mRNAs and cancer risk miRNAs.

The WGCNA(Weighted gene co-expression network analysis)^40^ method constructs a network based on the mRNA expression level of the system, showing the co-expression relationship between mRNAs. mRNAs with similar expression patterns may interact, jointly regulate a functional related pathway or affect different pathways. That is, if the expression trend of some mRNAs has the same change trend (expression pattern) with the change of different treatments, it can be considered that these mRNAs are likely to be enriched in a pathway or in mutually regulated pathways, so as to determine the core mRNAs in the whole network. Therefore, in this study, the expression data of differential mRNAs and miRNA were used to construct a new expression profile, and the expression profile was analyzed by WGCNA method. We then extracted the gene module with the highest correlation with cancer and P < 0.05 as the gene module for cancer. Cancer and normal phenotypes were used to identify cancer-related modules, and the differential mRNAs and miRNA in the modules were extracted as risk factors.

### Identifying cancer-related risk pathways in 21 cancer types

In order to explore the role of these risk mRNAs and risk miRNAs in the occurrence and development of cancer, we further conducted pathway enrichment analysis and identified pathways closely related to cancer in 21 cancer types. Based on risk mRNAs and miRNAs, we identified their significantly enriched pathways as risk pathways for each cancer type. The Cumulative hypergeometric test was used to calculate the significance of each pathway enriched by risk mRNAs and miRNAs. The formula of the cumulative hypergeometric test is as follows:

$${\rm{P}}=1-\mathop{\sum }\limits_{{\rm{k}}=0}^{{\rm{m}}}\frac{\left(\begin{array}{c}{\rm{n}}\\ {\rm{k}}\end{array}\right)\left(\begin{array}{c}{\rm{N}}-{\rm{n}}\\ {\rm{M}}-{\rm{k}}\end{array}\right)}{\left(\begin{array}{c}{\rm{N}}\\ {\rm{M}}\end{array}\right)}$$

Where N represents the number of background mRNAs (all genome-wide mRNAs), M is the number of a given pathway’s mRNAs and miRNAs that are annotated in the N mRNAs, n is total number of the risk mRNAs and miRNAs of a given cancer type, and m is the number of risk mRNAs and miRNAs in the given pathway. Pathways with P < 0.05 were considered risky pathways.

### Establishing the risk pathways’ crosstalk for 21 cancer types

Within each cancer type, we calculated the crosstalk between each pair of risk pathways based on the strength of the correlation between mRNAs and miRNAs. The Pearson product-moment correlation coefficient and unpaired t-test were used to measure the strength of the correlation between each of the two interrelated pathways. For mRNAs and miRNAs that appeared in both pathways m and n, we calculated their correlation strengths only if they interacted with other mRNAs or miRNAs in the PPI network. We then used the correlation strength to construct and assess the crosstalk of risk pathways. The formula for calculating the correlation strength is as follows:

$$\begin{array}{l}\mathrm{CS}({\rm{a}},{\rm{b}})={\rm{F}}({\rm{P}}({\rm{a}}),{\rm{P}}({\rm{b}}){\mathrm{| EXP}}_{{\rm{a}}},{\mathrm{EXP}}_{{\rm{b}}})\\ \quad \quad \quad \quad =-1\ast ({\log }_{{\rm{e}}}{\rm{P}}({\rm{a}})+{\log }_{{\rm{e}}}{\rm{P}}({\rm{b}})+{\log }_{{\rm{e}}}{\rm{P}}({\rm{a}},{\rm{b}}))\end{array}$$

where a is the mRNA that is annotated in pathway m; b is the mRNA that is annotated in pathway n; *EXP*_*a*_ and *EXP*_*b*_ are the expression values of mRNAs a and b in samples, respectively; P(a) and P(b) are the differential significance p-values of mRNAs a and b calculated using the unpaired Student’s t-test, respectively; and P(a,b) is the significant p-value of expression correlation coefficient between m and n mRNAs/miRNAs based on the Pearson’s product moment correlation coefficient.

Crosstalk of each pair of risk pathways was obtained by adding up all correlation strengths between them. Crosstalk of risk pathways m and n was calculated according to the following formula:

$${\mathrm{Crosstalk}}_{({\rm{i}},{\rm{j}})}=\mathop{\sum }\limits_{{\rm{i}}}^{{\rm{n}}}\mathrm{CS}$$

where n presents the number of all mRNA–mRNA and mRNA–miRNA interactions between any two pathways.

### Screening single therapeutic agents for 21 cancer types

Studies have shown that crosstalk between signaling pathways plays an important role in the occurrence and development of cancer. Therefore, evaluating the effect of drugs on pathway crosstalk based on the expression of drug targets can help to optimize the treatment of various cancers. From this perspective, to assess the effects of drugs on the dysfunctional crosstalk network, for each drug, we first removed its target mRNAs from the risk pathway. Next, we recalculated the crosstalk of risk pathways to quantify the destructive effects of drugs on the crosstalk network. At the same time, a method was designed and developed. Use the following formula to calculate the destructive score (DS) of drugs on crosstalk:

$$\mathrm{DS}({\rm{d}})=1-\frac{{\mathrm{Crosstalk}}_{{\rm{d}}}}{{\mathrm{Crosstalk}}_{{\rm{s}}}}$$

Where *crosstalk*_*d*_ represents the total Crosstalk after drug action; *crosstalk*_*s*_ represents the total Crosstalk in the cancer before removing the drug target. Drugs with DS > 0.01 were considered as single therapeutic meaningful drugs.

### Optimizing the combination of anticancer drugs for 21 cancer types

Based on the single drugs screened in the previous step, drugs with a DS value greater than 0.01 were selected for combination. Then, the targets corresponding to the drug combination in the risk pathway are removed to obtain the specific crosstalk network targeted by the drug combination. Risk pathway-specific crosstalks were then recalculated to measure the extent to which drug combinations disrupted the crosstalk network. Finally, compare the destructive score of the drug combination with the sum of the destructive scores of the two drugs, and select the combination whose drug combination is greater than or equal to the sum of the individual drugs. Through K-means clustering, disease samples were divided into high-risk group and low-risk group to construct survival models, so as to select the best combination drug. Drug combinations with P < 0.05 were considered to be of survival significance.

### Supplementary information

Supplementary Information

Supplementary Figure 1

## Data Availability

Among the input data processed during the reanalysis, the expression data of mRNA and miRNA were obtained from the TCGA database (http://tcga-data.nci.nih.gov/)^29^. miRNA target gene data come from miRTarBase (http://miRTarBase.cuhk.edu.cn/)^30^, mir2Disease (http://www.mir2disease.org/)^31^, miRecord (http://mirecords.biolead.org/)^32^, and TarBase (http://microrna.gr/tarbase)^33^. The protein-protein Interaction (PPI) network data used in this study came from HPRD (Human Protein Reference Database)^34^. Pathway data were obtained from the KEGG database^35^. Drug target data are from DrugBank^36^ and SM2miR^37^. Anticancer drug data comes from NCI (NATIONAL CANCER INSTITUTE) (https://www.cancer.gov/about-cancer/treatment/drugs).

## Code availability

The R codes used for Differential analysis, identification of risk genes and risk pathways, calculation of cross talk between risk pathways, individual drug screening, optimization of drug combination are available in https://github.com/ouqiyjl/oDrugCP/tree/main.
