# Supplementary material for: Optimizing drug combination and mechanism analysis based on risk pathway crosstalk in pan cancer
Source: Sci Data. 2024 Jan 16;11:74. doi: 10.1038/s41597-024-02915-y (PMC10791624; doi:10.1038/s41597-024-02915-y)
Supplement: Supplementary file 1 — Supplementary Information [file 41597_2024_2915_MOESM1_ESM.docx]

**Supplementary information**

**Supplementary Figure Legend**

**Supplementary Figure 1.** The confirmation of drug combination in 4 databases. The combination of the horizontal and vertical axes represents drug combinations. Each point represents a combination confirmed in the database. The shape of the points corresponds to different drug combination databases, and the color of the points represents the cancer type we predict the drug combination is effective for.

**Supplementary Table**

**Supplementary Table 1** Differentially expressed factors in 21 cancers

**Supplementary Table 2** Risk factors in 21 cancers

**Supplementary Table 3** Risk pathways in 21 cancers

**Supplementary Table 4** Pathway crosstalks in 21 cancers

**Supplementary Table 5** Drug combinations in 18 cancers

Supplementary Tables are available in https://github.com/ouqiyjl/oDrugCP/tree/main.
