# Supplementary figures and images for: Optimizing drug combination and mechanism analysis based on risk pathway crosstalk in pan cancer
Source: Sci Data. 2024 Jan 16;11:74. doi: 10.1038/s41597-024-02915-y (PMC10791624; doi:10.1038/s41597-024-02915-y)

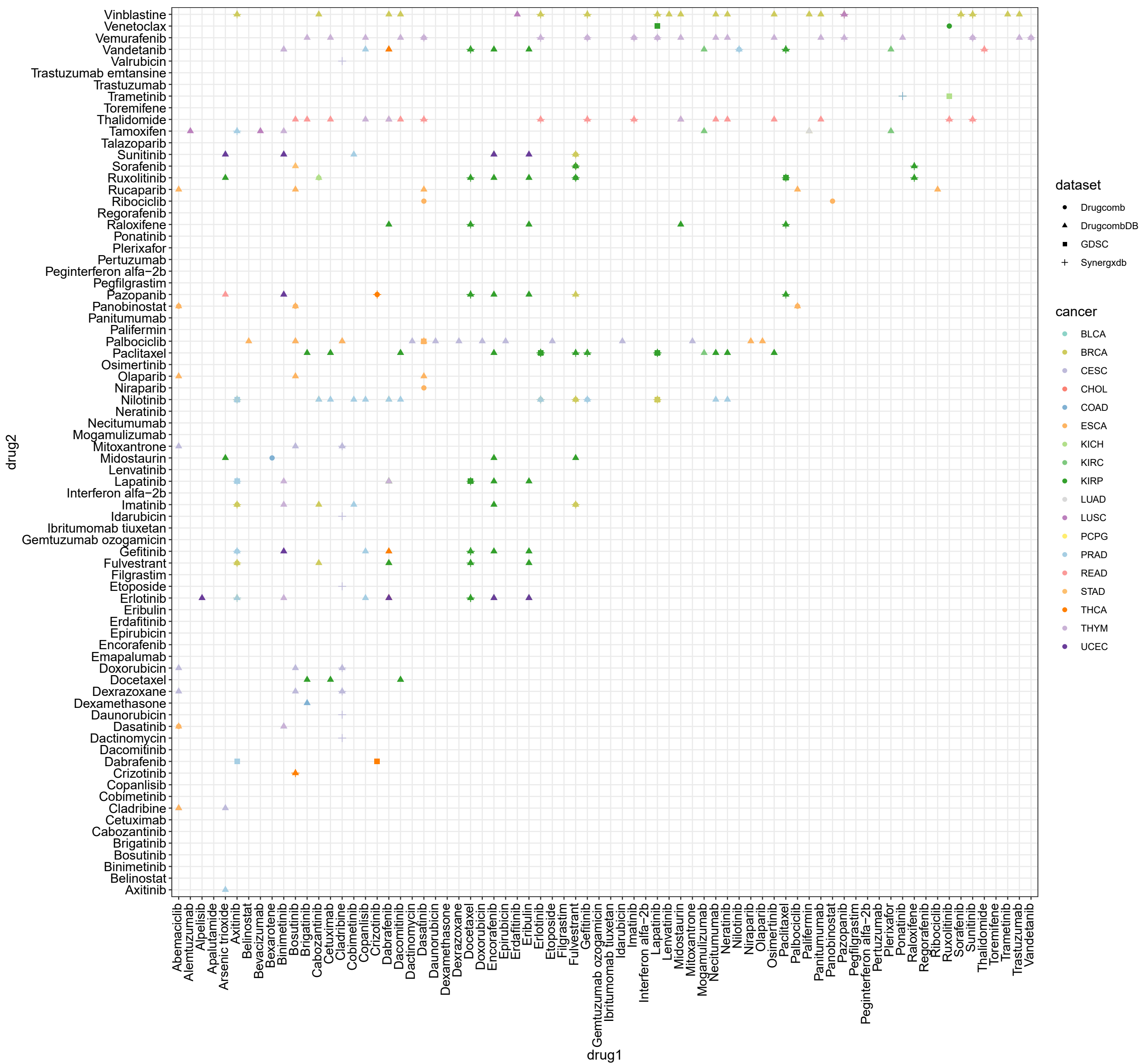

Supplement: Supplementary file 2 — Supplementary Figure 1 [file 41597_2024_2915_MOESM2_ESM.pdf]
